# Caution in Searching for Poisons

**Published:** 1855-11

**Authors:** 


Caution in Searching for Poisons.—Dr. Buchner observes that in the search for arsenic, while mere traces, only distinguishable by the most delicate tests, may be discoverable in the stomach and upper part of the intestinal canal, the poison may be often easily detected in the lower part of the canal, and that in proportion as the rectum is approached as also in the liver.—(*Report. fur Pharm.* 1855. No. 3.) *Ibid.*

